# Sheds for life: health and wellbeing outcomes of a tailored community-based health promotion initiative for men’s sheds in Ireland

**DOI:** 10.1186/s12889-022-13964-6

**Published:** 2022-08-20

**Authors:** Aisling McGrath, Niamh Murphy, Tom Egan, Noel Richardson

**Affiliations:** 1 School of Health Sciences, Waterford Institute of Technology, X91 K0EK Waterford, Ireland; 2 School of Business, Waterford Institute of Technology, X91 K0EK Waterford, Ireland; 3 National Centre for Men’s Health, Institute of Technology Carlow, R93 V960 Carlow, Ireland

**Keywords:** Men’s sheds, Men’s health promotion, Community, Evaluation, Gender-specific, Implementation, Physical activity, Mental health

## Abstract

**Background:**

Gender is increasingly recognised as a critical factor in designing community-based health promotion programmes. Men’s Sheds (‘Sheds’) are community-based informal environments that represent a safe space in which to engage cohorts of hard-to-reach (HTR) men in health promotion. Sheds for Life (SFL), the first structured health promotion initiative evaluated globally in Sheds, is a 10-week initiative co-designed with Shed Members (Shedders) and delivered directly in the Shed setting in Ireland. This research describes the health and wellbeing outcomes experienced by SFL participants.

**Methods:**

Purposive sampling was used to recruit a diverse representation of Shedders (*n* = 421) participating in SFL alongside a wait list control (*n* = 86). Questionnaires assessing constructs of health and wellbeing were administered one-to-one in Sheds at baseline, 3, 6 and 12 months. Descriptive data for health outcomes were generated for each time point and assessed for significant changes using inferential testing, while considering COVID-19 impact.

**Results:**

Outcomes related to subjective wellbeing, mental wellbeing, physical activity, social capital and healthy eating significantly increased post SFL (*p* < 0.05). Mental wellbeing scores (SWEMWBS) post SFL remained significantly higher than baseline despite COVID-19 impact (*p* < 0.05). Binary logistic regression indicated that the odds of a meaningful SWEMWBS change was significantly higher for shedders that had lower SWEMWBS (OR 0.804), less loneliness (OR 0.638) and lived alone (OR 0.456) at baseline. Shedders with lower SWEMBWS had higher odds of experiencing positive changes in life satisfaction (OR 0.911) and trust (OR 0.928), while Shedders who lived alone had also higher odds of experience positive changes in healthy eating (OR 0.481). Finally, inactive Shedders at baseline had higher odds of experiencing increased levels of physical activity (OR 0.582).

**Conclusions:**

Findings suggest that the inclusive, community-based SFL model is effective in engaging Shedders and facilitating positive and sustained changes in health and wellbeing outcomes. Using gender-specific approaches in the informal and safe environment of the Shed are effective in engaging men in structured health and wellbeing initiatives, particularly those who may be more vulnerable, isolated or lonely.

**Trial registration:**

This study has been retrospectively registered with the ‘International Standard Randomised Controlled Trial Number’ registry (ISRCTN79921361) as of 05/03/2021.

## Background

### Tailoring health promotion to men

In response to an unequal burden of ill-health and mortality in men, global health conversations and policies are increasingly calling for gender-specific health promotion strategies that target lifestyle and health behaviour change, particularly to so called ‘hard-to-reach’ (HTR) groups of men i.e. men who tend to be more isolated from, or reticent about accessing formal health services or social support networks due to geography, experiences of mental health issues, unemployment or changes in life course [1–4]. Indeed, recent responses have focused on the underlying factors that contribute to men’s avoidance of health promotion and health systems and on developing strategies to address these [5]. This has been driven by a growing body of evidence that advocates for a greater understanding of how gender intersects with economic, political, environmental and social determinants of health to influence men’s exposure to risk factors and health engagement [1, 6]. In particular, understanding the complexities of how masculinities are constructed through men’s engagement with health systems has focused attention on the need for gendered approaches towards engaging men with health at both policy and programme level [7, 8]. This approach requires a specific focus on tailored and targeted interventions that encourages sustained engagement by men [9, 10]. Evidence suggests that men who feel required to align with dominant traits of masculinity are less likely than women to; perceive themselves at risk for illness; believe they have internal control over their health; contemplate changing unhealthy habits; and utilise health care [1]. Barriers towards male help seeking are largely influenced by gendered practices and behaviour that conflict with reasons to seek help, as well as poor communication by health care professionals which can result in negative experiences of health services [11–14]. Moreover, within health-care systems, unconscious gender biases and heuristics based on gender stereotypes all affect engagement with health, resulting in differential health outcomes for men, women and gender minorities [12]. It is important that health initiatives: i) move away from a ‘one size fits all’ approach, ii) do not view men as a homogenous group, and iii) adopt a flexible approach to engage with different subpopulations of men [15]. Indeed, understanding how gender shapes men’s health practices is a critical first step in developing effective health promotion strategies that appeal to men [1]. Responses require recognition that the burden of ill health in men is caused by multiple complex factors that are exacerbated for socially disadvantaged and HTR cohorts [16]. There is an urgent need to address a key paradox in men’s health, whereby men who are most in need of intervention are least likely to engage with health services. The task for men’s health promotion is to reach beyond the ‘worried well’ by designing innovative and tailored programmes targeted to specific sub-population groups of HTR men [17, 18].

### The Men’s sheds as a setting for health promotion

Promoting health within the community setting enables the creation of supportive environments and the potential to encourage positive behaviour change through a focus on equity, inclusion and social coherence [19]. In the case of men’s health promotion, the community setting is conducive to a gender-sensitive environment that can facilitate a strengths-based, multi-sectoral approach in a non-clinical and informal atmosphere where men feel safe [20, 21]. Research has demonstrated that service providers can maximise the reach of interventions targeting at-risk cohorts of men in community settings through partnership and gender sensitised recruitment strategies anchored within community groups [22]. Indeed, a range of community-level men’s health initiatives that incorporate gender into their design and delivery have demonstrated the efficacy of this approach [23–28]. The rise in popularity of Men’s Sheds (‘Sheds’) is based on their community-based and non-clinical nature, alongside the sense of purpose, social support, camaraderie and reciprocity offered within the socially acceptable and masculine environment of the Shed [29]. Sheds are autonomous, Shed member (‘Shedder’)-sustained, grassroots organisations that provide a space for men to socialise, work on projects, share goals and develop new skills [30, 31]. Originating in Australia in the 1980s, Sheds have flourished organically in Ireland since their arrival in 2011 and are testament to a need for men to identify with a space that facilitates meaning, social support, safety and belonging [3, 32]. Moreover, due to their organic and informal nature, research has found that Sheds appeal to HTR groups of men [33, 34]. Sheds are variable spaces in terms of physical size, location type and membership where activities consist of a focus on a primary utility function as well as social participation [35]. These include activities such as woodworking, mechanics, bee keeping, gardening, art, music and card playing. While activities in different Sheds may vary, they offer men a sense purpose through a focus on work, independence and safety and respite within a male focused space [36]. Although Sheds appeal to predominantly retired men, they have demonstrated their inclusivity for men from diverse backgrounds and varying abilities, also offering opportunities for intergenerational learning [32, 37, 38]. Although, Sheds are not explicitly considered health interventions [35], research has demonstrated Shedders’ agency in organising health promotion activities within Sheds such as mental health and prostate cancer talks, demonstrating a willingness to engage with health and wellbeing in Sheds [39, 40]. Based upon their inherent health promoting qualities and ready access to men who may be reticent to engage with traditional health services, Sheds represent an attractive setting in which to build structured health initiatives. However, caution is warranted when attempting to fuse formal health promotion with the informal Shed environment. Indeed, research has highlighted this informal space as an integral element to the inherent health promoting qualities of Sheds, and that efforts to provide pathways for Shedders to access support should not compromise the integrity of Sheds as peer run spaces, as to do so may be damaging to Shedders wellbeing and Shed ethos [3, 31, 41].

### Sheds for life – health promotion ‘for Shedders by Shedders’

The first iteration of a health promotion programme for Sheds in Ireland was developed by the Irish Men’s Sheds Association (IMSA) in 2016. ‘Sheds for Life’ (SFL) emanated from calls at a research, service provider (SP) and Shedder level to deliver health promotion in Sheds. This resulted in the piloting of a range of discrete health promotion initiatives in Sheds alongside scoping work which sought to reach a consensus on an acceptable and respectful approach to deliver SFL in Sheds [3]. Over time, SFL evolved into a partnership network comprising the IMSA, academics, an advisory group (consisting of men’s health promotion specialists and twelve allied SP organisations, along with Shedder representation. Sheds for Life is overseen by the IMSA and delivered by the SPs directly in the Sheds. It delivers targeted and tailored wellbeing and life skill components and has been co-designed with Shedders to ensure a respectful and appropriate delivery model. Sheds for Life is delivered over 10-weeks in the Sheds and commences with a health check for participants. It consists of three other core components of weekly physical activity sessions (walking or chair-based strength and mobility exercises), weekly healthy eating sessions for six weeks (Healthy Food Made Easy; HFME; a nutrition and cooking programme endorsed by the health services in Ireland) and a mental health workshop which aligns with the key pillars of Ireland’s national men’s health policy ‘Healthy Ireland Men’ [16]. Several optional components are also available into which Sheds can self-select, aligning with the needs of Shedders (e.g. diabetes, cancer and oral health awareness, suicide prevention training, cardiopulmonary resuscitation training (CPR), digital literacy). A detailed outline of the evolution, content and structure of SFL from its conception to a 10 week programme is available in a protocol paper [42].

The underpinning vision of SFL is to normalise conversations about health and wellbeing in Sheds and encourage help seeking among Shedders. This vision potentially conflicts with traditional norms of masculinity that are characteristic of more HTR groups of men [1]. Central to this approach is the positioning of Shedders as key decision makers alongside SP organisations, researchers and the IMSA as part of a participatory research approach [42]. Established implementation frameworks [43–45] are used in applying principles of implementation science to guide the implementation and evaluation of SFL. These frameworks guide the engagement process in the implementation of SFL across the variable environment of the Sheds as well as accounting for the interaction between Sheds and SP organisations. They play an important role in facilitating acceptability and optimising recruitment, participation and engagement in SFL. The design and delivery of SFL draws heavily on gender-specific approaches layered upon the male-specific, safe, familiar environment and sense of social support inherent in Sheds. These approaches are outlined in detail elsewhere [42], but notably involve; informal and interactive delivery, removal of costs barriers, trust building, the use of a comprehensive health check and non-typical health related components to engage men, and tailoring the SFL programme to each Shed to provide a sense of autonomy and control for Shedders. The study adopts a hybrid effectiveness-implementation design to consider the effectiveness of its implementation in order to promote translation into the real world context from the outset, while simultaneously understanding the impact of SFL on Shedders wellbeing [42, 46, 47].

This particular research study focuses on the impact of the SFL initiative on the health and wellbeing outcomes of participants. Specifically, SFL targets health and wellbeing outcomes related to; subjective wellbeing (e.g. life satisfaction, life worth and self-rated health), health behaviours (physical activity, propensity to seek health information, diet and cooking skills), social capital (belonging, close support and trust), self-efficacy, and mental health. Understanding the efficacy of SFL in enhancing the health and wellbeing outcomes of Shedders will be critical to justification of scale-up. Moreover, while a body of research has highlighted the potential of Sheds to be health enhancing [30, 33, 35, 48, 49], there remains limited high-quality or empirical research evidencing the impact of health promotion initiatives conducted in the Shed setting [32, 49]. This has been a noted limitation in assessing the Shed-health link. Further research is therefore needed in this area to demonstrate the impact of more formal health promotion programmes conducted in the Shed setting among Shedders. Furthermore, while there have been calls to deliver more structured health promotion in Sheds, there has been no evidence to date of any formal evaluation of such endeavours. The aim of this research therefore is to determine the impact of SFL on the health and wellbeing outcomes of participants – the first formally evaluated health promotion initiative in Sheds.

## Methods

This study evaluates the effectiveness of SFL on the health and wellbeing outcomes of participants. A detailed protocol outlining the study design as well as the implementation approach is available [42].

### Participants and sampling

Respecting the autonomous and informal environment of the Sheds [3], purposive sampling by way of an expression of interest process was used to recruit Sheds to participate in SFL. The sampling process endeavoured to incorporate a diverse representation of Sheds (large/small, urban/rural). Sheds that expressed an interest in participating were then visited by the first author and members of the IMSA to discuss the SFL process and to recruit individual Shedders. This purposive sampling approach was effective in reaching men in the familiar setting of the Shed creating a sense of acceptability and building trust and rapport between Shedders and the SFL team. In total, 31 Sheds out of a potential 44 across the selected counties opted into SFL – a response rate of 70%. Data were collected at the recruitment phase to establish the reach of SFL by identifying the number of Shedders who regularly attended the participating Sheds. It was estimated that 565 of participating Shed members were active members at the time of recruitment, with the majority of these (*n* = 421; 75%) opting to participate in SFL and the supporting evaluation. An assessment at baseline of the population who opted into SFL also suggested that this cohort of Shedders met the criteria of being HTR in terms of factors such as; being older, out of work, with lower education and risk of isolation (see results; ‘profile of Shedders’) [34]. This suggested that the recruitment strategy was effective in engaging the target group [34, 50]. Inclusion criteria comprised all adult males who were active Shed members, had a good proficiency in the English language, and could give informed consent. Due to capacity and resource constraints of SPs along with the capriciousness of Shed environments, SFL was implemented on a phased basis across two cohorts, each consisting of two counties in Ireland between March and May 2019 and September to November 2019 (for further detail on these areas see protocol [42]). During the course of delivery in the first cohort (*n* = 12 clusters; *n* = 212 Shedders), a wait list control cohort served as a comparator (*n* = 3 clusters; *n* = 89 Shedders), and these were a subset of the second cohort (*n* = 9 clusters, *n* = 209 Shedders). During Shed visits, all participants had the details of the research clearly explained to them through verbal and written instruction and informed written consent was obtained by a member of the research team. Further details on consent, ethics and data management are available elsewhere [42]. The study received ethical approval from Waterford Institute of Technology Research Ethics Committee (REF: WIT2018REC010). This study has also been registered with the ‘International Standard Randomised Controlled Trial Number’ registry (ISRCTN79921361).

### Data collection

Questionnaires were administered to participants by the researcher one-to-one in the Shed setting to account for potential literacy issues, limit missing data and build rapport and trust between the researcher and Shedders. Due to the informal nature of Sheds, absence of data does not necessarily indicate drop out from SFL, rather due to constraints associated with research capacity, specifically in terms of aligning data collection with Shed opening hours, follow up rates vary and rescheduling of data collection was not possible. Questionnaires were administered at baseline (T1), 3 M (T2: post the 10 week intervention), 6 M (T3) and 12 months (T4) to Cohort 1 (C1) and Cohort 2 (C2). During T3 and T4 participants in C2 were actively experiencing COVID-19 restrictions and Sheds were closed due to the pandemic. Therefore, questionnaires were administered via phone in order to promote participant retention and complete follow-up to 12 months. The impact of COVID-19 will therefore be considered in the results where relevant. A range of participant demographics were recorded at baseline such as date of birth, living arrangements, marital status, educational attainment, employment status, ethnicity, length of Shed membership and frequency of attendance. Core health and wellbeing outcomes measured at all-time points up to T4 included subjective wellbeing and help seeking, lifestyle measures (physical activity (PA), physical activity self-efficacy, alcohol, smoking, and diet), mental wellbeing and social capital. Self-rated health (SRH) was measured using a single question Likert scale with high reliability among older men [51]. The single-item PA measure was used to record PA levels [52]. The Self-Efficacy for Exercise (SEE) scale was used to measure physical activity self-efficacy [53]. Life worth and satisfaction were recorded using the Office of National Statistics subjective wellbeing 11-point scales [54]. Mental wellbeing was measured using the Short Warwick-Edinburgh Mental Wellbeing Scale (SWEMWBS) with raw to metric score conversion where a change of 2+ was clinically meaningful [55], along with constructs assessing changes in mental health perceptions. Loneliness was measured at all-time points using the UCLA 3-item scale [56]. Social Capital was measured based on relevant recommendations from WhatWorksWellbeing [57], capturing trust, belonging and close support. Interpersonal trust was measured using the Office of National Statistics 11-point scale [54]. Participants were asked about their levels of daily fruit and vegetable consumption, cooking style, cooking frequency and willingness to cook. Confidence constructs around cooking and food preparation were measured via a 12 item scale [58]. Constructs included assessment of cooking using raw ingredients, following a simple recipe, planning meals before shopping, shopping for food on a budget, shopping for healthier food to eat, cooking new foods, cooking healthier foods, storing food safely, using leftovers to cook other meals, cooking whole raw chicken from scratch, and reading food labels and food hygiene. The six dimensions (physical functioning,social functioning, pain, role limitation, vitality and mental health) of the SF-6D were also assessed up to T4 to assess change in utility scores [59]. Utilities are preference weights, where preference can be equated with value or desirability and are measured on a cardinal scale of 0–1 where 0 indicates death and 1 indicated full health [60] (See protocol paper for more detailed information on instruments used [42]). Due to constraints associated with research capacity, specifically in terms of aligning data collection across multiple locations with a small research team and Shed opening hours with sporadic attendance, rescheduling of data collection was not possible and follow up rates varied.

### Data analysis

Questionnaire data were analysed using Statistical Packages for the Social Sciences (SPSS V 25). Descriptive statistics were generated for each variable. Intervention effect on health and wellbeing outcomes were determined by comparing measures across time periods using paired samples t-tests and repeated measures ANOVA models. Differences between the intervention group (IG) and control group (CG) as well as between C1 and C2 at different time points were determined via independent-samples t-tests. Data were analysed to assess measures from T1 to T2, immediately following the 10-week SFL intervention. Further data was then analysed to assess measures from T1 at T3 and T4. This analysis involved separating C1 and C2 as C1 did not experience COVID-19 restrictions at T3 and T4 whereas C2 were actively experiencing COVID-19 restrictions. The aim of this approach was to highlight the potential impact of COVID-19 on the results. An earlier publication from this study [61] outlines the impact of COVID-19 on SFL participants in further detail and also highlights similarities between the groups prior to COVID-19. Therefore C1 and C2 were only analysed separately as subsets of the IG for T3 and T4. Following this analysis, a binary logistic regression model was used to control for various factors when assessing the significance of the relationship between these factors. The selection of the dependent and independent variables was guided by previous work and specific patterns that emerged from the data [34]. Covariates were selected for each regression model based on stepwise selection [62], and previous work that highlighted relevant variables such as age, education, living situation, physical activity levels and baseline mental wellbeing as impacting on the respective dependent variable [14, 26, 34, 58, 63–68] as well as trends that emerged during data analysis across time points. For example, literature suggests that variables such as age, gender and education impact on levels of life satisfaction [63, 64], while further studies cite the role of living situation and loneliness as impacting on levels on mental wellbeing [33, 69]. Regression analysis as a multivariate technique examines more than two variables at a time and allows assessment of significant relationships while controlling for other variables. Changes between these variables from baseline (T1) to 3 months (T2) were used for this analysis as the SFL intervention lasted for 10-weeks, and because measures at 3 months were not subject to COVID-19 as a confounder as highlighted in Table 2. The chosen regression type was binary logistic regression and the dependent variables used were; (a) Positive Change in life satisfaction from T1 to T2 (0 = No, 1 = Yes); (b) Positive change in SWEMWBS above a threshold gain of 2 (0 = No, 1 = Yes), as this change is considered clinically meaningful [55]; (c) Positive change in weekly PA of 1 plus day(0 = No, 1 = Yes); (d) Positive change in food preparation and cooking confidence (0 = No, 1 = Yes); and (e) Positive change in trust (0 = No, 1 = Yes). For such regression models, the interpretation is typically of odds ratios and this varies depending on whether the independent variables (IV) are categorical or continuous - with odds ratios of greater than 1 indicating events that are more likely to occur as the predictor increases and vice versa.

## Results

### Reach and follow-up rates

The overall estimated reach of SFL was 74.51% based on the numbers who enrolled (*n* = 421) compared to those who were eligible to enrol from participating Sheds (*n* = 565). Mean percentage attendance rates of individual components were calculated by combining data on attendance rates gathered via SPs attendance records and participants’ self-reported data. The average attendance rates across the 10 weeks for the core components of PA, mental health and HFME were 73.0, 72.86 and 73.2% respectively. Of those signed up to participate in cancer, diabetes and oral health awareness, the rates were 73.45, 74.0 and 62.1% respectively. Of those who participated in suicide prevention training, CPR and digital literacy the rates were 73.0, 76.2 and 61.6% respectively. Some 384 participants (91.2%) completed the health check at the commencement of SFL. Questionnaires were administered at T1 (*n* = 383), T2 (*n* = 229), T3 (*n* = 211) and T4 (*n* = 285), demonstrating an average follow up rate of 63.1%.

### Profile of shedders

The baseline characteristics of SFL participants (*n* = 384), including demographics as well as objective and subjective health measures, have been described in detail elsewhere [34]. Overall the results highlighted that a majority of this population of Shedders were over 65 years (77.2%) with a mean age of 69.1 ± 9.14 within a range of 27–90 years. Almost a quarter (24.9%) of participants had no more than primary education with the majority (77%) having some secondary level education. Most participants were not currently in employment (88.6%; 80.4% retired and 8.2% unemployed or unable to work), while the majority were married (74.2%), with over a quarter (25.8%) separated or divorced and 17.8% living alone. In terms of health status, an earlier publication from this study [34] has indicated that while the majority of Shedders rate their subjective wellbeing in positive terms, their objective health measures place them in an ‘at-risk’ category in terms of hypertension (84.1%), overweight and obesity (86.8%) and high waist circumference (78.3%), with the majority (68.2%) physically inactive. Following their initial health check, the vast majority of Shedders (79.6%) were referred to their GP based on a parameter of concern arising from the health check results. Further detail on baseline characteristics of this cohort at baseline can be found in previous work [34].

### Initial impact of SFL (baseline to 3 months)

Table 1 highlights changes in wellbeing and lifestyle outcomes up to 3 months, immediately following the SFL 10-week programme in the IG compared to the CG, along with significance testing. It also shows mean *p* values in differences between the IG and CG at T1 and T2. While significance testing at baseline between these groups may be viewed as superfluous by some researchers [70], it was decided that since the groups had not been randomised, mean *p* values ought to be displayed at T1 to demonstrate similarities between groups. Table 1 demonstrates that the vast majority of variables at T1 were similar in values when comparing the IG and CG and, consequently, no significant differences were found between these variables at baseline. At T2, following the SFL intervention, there is a clear divergence in values between the two groups with IG experiencing significant positive improvements compared to largely maintained values in the CG from T1 to T2. Some of the notable trends from T1 and T2 include:

**Table 1:** Wellbeing and lifestyle outcomes up to T2 with control comparison

	T1		***p***-value (IG vs CG at T1)	T2		***p***-value (IG vs CG at T2)
	IG (Mean ± SD (*n* = 382)	CG (Mean ± SD (*n* = 87)		IG (Mean ± SD (*n* = 236)	CG (Mean ± SD (*n* = 75)	
Life Satisfaction	7.98 ± 1.71	7.76 ± 1.71	0.285	8.56 ± 1.45**	7.69 ± 1.64	0.000 *
Life Worth	8.20 ± 1.61	7.91 ± 1.66	0.063	8.89 ± 1.29**	7.93 ± 1.72	0.000*
Self-rated health [*1 = Excellent 5 = Poor]*	2.84 ± 0.96	3.18 ± 0.91	0.002 *	2.54 ± 0.94**	2.92 ± 0.94**	0.003 *
Like finding out about one’s health [*1 = Often 4 = Never]*	1.82 ± 0.84	1.83 ± 0.87	0.910	1.43 ± 0.70**	1.77 ± 0.92	0.001*
Mental Wellbeing (SWEMWBS)	26.78 ± 4.89	26.94 ± 4.77	0.776	30.81 ± 4.79**	26.62 ± 4.93	0.000 *
Depression Prevalence [*1 = probable depression 4 = high mental wellbeing]*	3.34 ± 0.70	3.40 ± 0.63	0.466	3.73 ± 0.53**	3.35 ± 0.73	0.000 *
Certainty managing mental health [*1 = very certain 5 = very uncertain]*	2.11 ± 0.96	2.09 ± 0.90	0.831	1.38 ± 0.72**	2.16 ± 0.98	0.000 *
Comfort conversing about mental health [*1 = very certain 5 = very uncertain]*	2.11 ± 0.96	2.09 ± 0.90	0.831	1.38 ± 0.72**	2.16 ± 0.98	0.000 *
Feel equipped with mental health supports [*1 = very certain 5 = very uncertain]*	2.37 ± 1.08	2.33 ± 1.07	0.744	1.44 ± 0.72**	2.33 ± 0.99	0.000 *
Loneliness (3-item UCLA)	3.31 ± 0.89	3.25 ± 0.79	0.602	3.40 ± 0.97	3.32 ± 1.05	0.539
Social Capital Trust	6.82 ± 1.98	6.38 ± 1.64	0.053	7.51 ± 1.87**	6.44 ± 1.42	0.000 *
Social Capital: Belonging [*1 = Strongly Agree 4 = Strongly Disagree]*	1.32 ± 0.54	1.22 ± 0.44	0.120	1.11 ± 0.33**	1.12 ± 0.34	0.634
Social Capital: Close support [*1 = Strongly Agree, 4 = Strongly Disagree]*	1.29 ± 0.51	1.35 ± 0.61	0.355	1.11 ± 0.35**	1.27 ± 0.50)	0.002 *
Utility Scores *[Scale 0–1, higher scores indicate improved utility]*	0.79 ± 0.12	0.78 ± 0.12	0.195	0.83 ± 0.10**	0.79 ± 0.13	0.005 *
Days of Week Physically Active	3.07 ± 2.57	2.83 ± 2.40	0.434	4.32 ± 2.86**	3.09 ± 2.49	0.001 *
Days walking per week for ≥10 mins	4.14 ± 2.78	4.14 ± 2.39	0.997	5.78 ± 2.29**	4.05 ± 2.46	0.000 *
Physical activity self-efficacy	53.17 ± 20.99	52.54 ± 21.23	0.801	64.85 ± 19.67**	50.72 ± 22.15	0.000 *
Meeting Physical Activity Guidelines	32%	29%	0.732	47%**	36%	0.000 *
Portions of Daily Fruit & Vegetables	3.36 ± 1.76	3.15 ± 1.71	0.310	3.88 ± 1.77**	3.27 ± 1.77	0.010 *
Cooking Habits [*1 = don’t cook at all, 4 = cook meals from scratch]*	2.83 ± 1.37	2.56 ± 1.39	0.100	3.12 ± 1.31**	2.52 ± 1.45	0.001 *
Cooking Frequency [*1 = Often 4 = Never]*	2.12 ± 1.13	2.14 ± 1.06	0.911	1.88 ± 1.01**	2.13 ± 1.09	0.076
Total cooking and food preparation confidence scores (higher scores indicates increased confidence)	33.16 ± 10.55	31.64 ± 10.98	0.232	39.30 ± 8.68 **	32.25 ± 10.79	0.000*
Alcohol Days per week	1.58 ± 1.71	1.44 ± 1.81	0.519	1.22 ± 1.59 **	1.42 ± 1.76	0.351
Alcohol Units per session	5.81 ± 8.08	4.83 ± 4.79	0.301	3.59 ± 3.97**	6.54 ± 8.60**	0.000*

*Difference between IG and CG is significant at *p* ≤ 0.05 ** Difference from T1 to T2 are significant at p ≤ 0.05 in IG and CG

*IG* Intervention group, *CG* Control group, T1 = Baseline T2 = 3 months

### Subjective wellbeing and mental health

There were significant increases in the IG in subjective wellbeing; life satisfaction, life worth and SRH; this compares to no significant changes in life satisfaction and life worth for the CG, although this group did experience a positive improvement in SRH. The IG also reported an increased propensity to seek information about their health following SFL (at T2), while there was no significant change in the CG, meaning that the difference between the two cohorts became significant at T2.

There was a significant improvement in Mental Wellbeing scores (SWEMWBS) for the IG at T2 and these scores were also significantly higher than that of the CG. Similarly, there was a positive decline in depression prevalence scores for the IG with Shedders in this cohort also reporting increased levels of certainty in managing their mental health, comfort having a conversation about mental health and feeling equipped with practical supports to maintain their mental health. There were no significant changes in these scores at T2 among Shedders in the CG who had not yet received the SFL intervention, while the differences between the IG and CG following SFL were significant. There were no significant differences in loneliness scores between the IG and CG at baseline or following SFL at T2.

Shedders trust ratings increased significantly for the IG at T2 compared to no change for the CG with a significant difference between groups at T2. In the IG, Shedders reported an increased sense of belonging to their Shed at T2. The CG did not experience this change although differences between groups were not significant at T2. Shedders sense of having close support significantly increased at T2 from baseline with no change for the CG and a significant difference between groups.

Utility scores which comprise the six dimensions of the SF-6D (physical functioning, social functioning, role limitation, vitality, mental health and pain) significantly improved for the IG compared to minimal change for the CG at T2 where differences between the two groups became significant. This is discussed in the context of programme cost effectiveness elsewhere [71].

### Physical activity and lifestyle

The number of days that Shedders were physically active significantly increased for the IG at T2 and was significantly higher than the CG at this time point. Prior to this, there were no significant differences between groups in terms of weekly PA. A similar trend can be seen in days spent walking for > 10 minutes, with significant increases occurring in the IG at T2 compared to no significant change for the CG. Similarly, the IG experienced a significant improvement PA self-efficacy from T1 to T2 compared to significant change in the CG. Alongside this trend, there was a significant increase in IG members meeting the PA guidelines at T2.

In relation to smoking, a minority of Shedders reported as current smokers (IG, 8.4%; CG, 13.8%). There were no changes in smoking rates at T2 and no significant differences between the IG and CG. A similar proportion of Shedders reported consuming alcohol (IG, 68.3%; CG, 67.8%) at baseline. There was a significant reduction in days per week spent consuming alcohol for the IG at T2 compared to no significant difference for the CG. Units of alcohol consumed per session also reduced significantly for the IG at T2 while increasing significantly for the CG, with a significant difference between the two groups at T2.

There was a significant increase in fruit and vegetable consumption between T1 and T2 for the IG while levels in the CG remained constant. There was also a significant increase in the number of Shedders reporting a positive change in their cooking habits (i.e. cooking meals from scratch vs using microwave ready meals) between T1 and T2 in the IG. No such change was observed in the CG with a significant difference in cooking habits occurring between the groups at T2. There was also a significant increase in cooking frequency in the IG. Total scores in relation to cooking and food preparation confidence significantly increased for the IG at T2, and were significantly different between the IG and CG at T2.

#### Medium-term impact of SFL (baseline through to 12 M)

In addition to T1 data collection which assessed for initial changes in health and wellbeing outcomes at 3 months following the 10-week SFL intervention, data collection was repeated at 6 months (T3) and 12 months (T4) to assess for potential maintenance of positive behaviour change following the intervention. Table 2 highlights the mean scores at T3 and T4 and assessed for significant changes from baseline (T1) in the IG. Considering that COVID-19 is a potential confounder in the results for C2 at T3 and T4, Table 2 also has a breakdown of both cohorts (C1 and C2) at these time points to account for this. COVID-19 was also a contributing factor to limitations in the control group and data for the CG is not available after 3 months; hence all data in Table 2 relates to the IG only.

**Table 2:** Wellbeing and lifestyle outcomes at T3 and T4 for the IG by Cohort

	T3				T4			
	IG (Mean *n* = 214)	C1 (Mean *n* = 69)	C2 (Mean(*n* = 145)	***p***-value (C1 v C2)	IG (Mean *n* = 272)	C1 (Mean *n* = 145)	C2 (Mean(*n* = 127)	***p***-value (C1 v C2)
Life Satisfaction	7.97 ± 1.66	8.76 ± 1.55 **	7.83 ± 1.69	0.065	8.00 ± 1.56	8.21 ± 1.33	7.77 ± 1.76	0.021
Life Worth	8.50 ± 1.49 **	8.54 ± 1.48 **	8.48 ± 1.46 **	0.781	8.42 ± 1.47	8.52 ± 1.34	8.30 ± 125	0.207
Self-rated health [*1 = Excellent 5 = Poor]*	2.51 ± 0.97**	2.12 ± 0.88 **	2.69 ± 0.96	0.000*	2.61 ± 0.93 **	2.12 ± 0.91 **	2.71 ± 0.94	0.910
Like finding out about one’s health [*1 = Often 4 = Never]*	1.33 ± 0.59**	1.25 ± 0.60**	1.37 ± 0.59**	0.171	1.52 ± 0.78**	1.52 ± 0.84**	1.53 ± 0.71**	0.944
Mental Wellbeing (SWEMWBS)	30.67 ± 4.46**	31.62 ± 5.00 **	30.23 ± 4.13 **	0.035 *	28.89 ± 4.63**	29.46 ± 4.21 **	28.24 ± 5.01 **	0.033 *
Depression Prevalence [*1 = probable depression 4 = high mental wellbeing]*	3.78 ± 0.53 **	3.77 ± 0.57 **	3.79 ± 0.51 **	0.835	3.62 ± 0.59 **	3.72 ± 0.52**	3.51 ± 0.64 **	0.006
Certainty managing mental health [*1 = very certain 5 = very uncertain]*	1.56 ± 0.74 **	1.61 ± 0.61 **	1.60 ± 0.77**	0.416	1.76 ± 0.92 **	1.72 ± 0.95 **	1.82 ± 0.88	0.387
Comfort conversing about mental health [*1 = very certain 5 = very uncertain]*	1.56 ± 0.77 **	1.62 ± 0.86 **	1.54 ± 0.75**	0.576	1.57 ± 0.82 **	1.57 ± 0.87 **	1.58 ± 0.75**	0.911
Feel equipped with mental health supports [*1 = very certain 5 = very uncertain]*	1.80 ± 0.93 **	1.58 ± 0.72**	1.88 ± 0.98 **	0.041 *	1.93 ± 1.03 **	1.89 ± 1.10**	1.88 ± 0.88 **	0.507
Loneliness (3-item UCLA)	4.13 ± 1.71 **	3.08 ± 0.51	4.62 ± 1.84 **	0.000 *	4.17 ± 1.79 **	3.51 ± 1.08	4.93 ± 2.11**	0.000 *
Social Capital Trust	7.35 ± 1.89 **	7.61 ± 2.01 **	7.23 ± 1.81	0.160	7.10 ± 1.86	7.27 ± 1.67 **	6.91 ± 2.05	0.119
Social Capital: Belonging [*1 = Strongly Agree 4 = Strongly Disagree]*	1.11 ± 0.37 **	1.10 ± 0.30 **	1.11 ± 0.39 **	0.868	1.14 ± 0.36**	1.11 ± 0.39 **	1.17 ± 0.40	0.124
Social Capital: Close support [*1 = Strongly Agree, 4 = Strongly Disagree]*	1.07 ± 0.27 **	1.03 ± 0.24 **	1.09 ± 0.28 **	0.135	1.14 ± 0.48 **	1.11 ± 0.39 **	1.18 ± 0.56	0.193
Utility Scores *[Scale 0–1, higher scores indicate improved utility]*	0.85 ± 0.09**	0.87 ± 0.87**	0.84 ± 0.97**	0.570	0.84 ± 0.09 **	0.84 ± 0.87**	0.83 ± 0.10 **	0.524
Days of Week Physically Active	3.58 ± 2.56**	3.84 ± 2.00 **	3.45 ± 2.78	0.300	3.77 ± 2.43**	3.45 ± 2.37	4.13 ± 2.45 **	0.240
Days walking per week for ≥10 mins	5.12 ± 2.34 **	4.75 ± 2.24 **	5.27 ± 2.37 **	0.128	4.87 ± 2.53 **	4.44 ± 2.43	5.34 ± 2.45 **	0.004 *
Physical activity self-efficacy	67.32 ± 17.34 **	65.91 ± 16.87**	68.02 ± 17.58 **	0.411	65.85 ± 21.79**	64.2 ± 20.19 **	66.99 ± 23.49**	0.430
Meeting Physical Activity Guidelines	39.4%**	40.6%*	38.9%	0.81	42.7**	37.4	48.8**	0.060
Portions of Daily Fruit & Vegetables	3.88 ± 1.72**	3.87 ± 1.74 **	3.89 ± 1.73 **	0.310	3.78 ± 1.60 **	3.69 ± 1.71 **	3.89 ± 1.47**	0.929
Cooking Habits [*1 = don’t cook at all, 4 = meals from scratch]*	3.15 ± 1.31 **	3.36 ± 1.18 **	3.04 ± 1.36 **	0.098	3.27 ± 1.23 **	3.31 ± 1.24 **	3.23 ± 1.23 **	0.674
Cooking Frequency [*1 = Often 4 = Never]*	1.92 ± 1.00	1.61 ± 0.89 **	2.06 ± 1.01	0.002 *	1.86 ± 0.99**	1.85 ± 0.98 **	1.90 ± 1.01 **	0.606
Total cooking and food preparation confidence scores (higher scores indicates increased confidence)	39.12 ± 9.61 **	42.35 ± 7.63 **	37.56 ± 10.07 **	0.001	39.13 ± 9.01 **	39.49 ± 8.68 **	38.73 ± 9.37 **	0.506
Alcohol Days per week	1.61 ± 1.68	1.26 ± 1.44	1.86 ± 1.81	0.290	2.09 ± 1.86**	2.18 ± 1.88	1.97 ± 1.83	0.466
Alcohol Units per session	4.36 ± 3.44 **	4.35 ± 3.44 **	4.38 ± 3.47**	0.000	6.30 ± 4.85	7.17 ± 5.28	5.05 ± 3.86 **	0.004

* Difference between C1 and C2 is significant

**Difference is significant from T1 for IG, C1 and C2

T3 = 6 months T4 = 12 months

*IG*Intervention group, *C1* Cohort 1 (pre-COVID cohort) C2 = Cohort 2 (COVID cohort)

### Subjective wellbeing and mental health

**Fig. 1:**
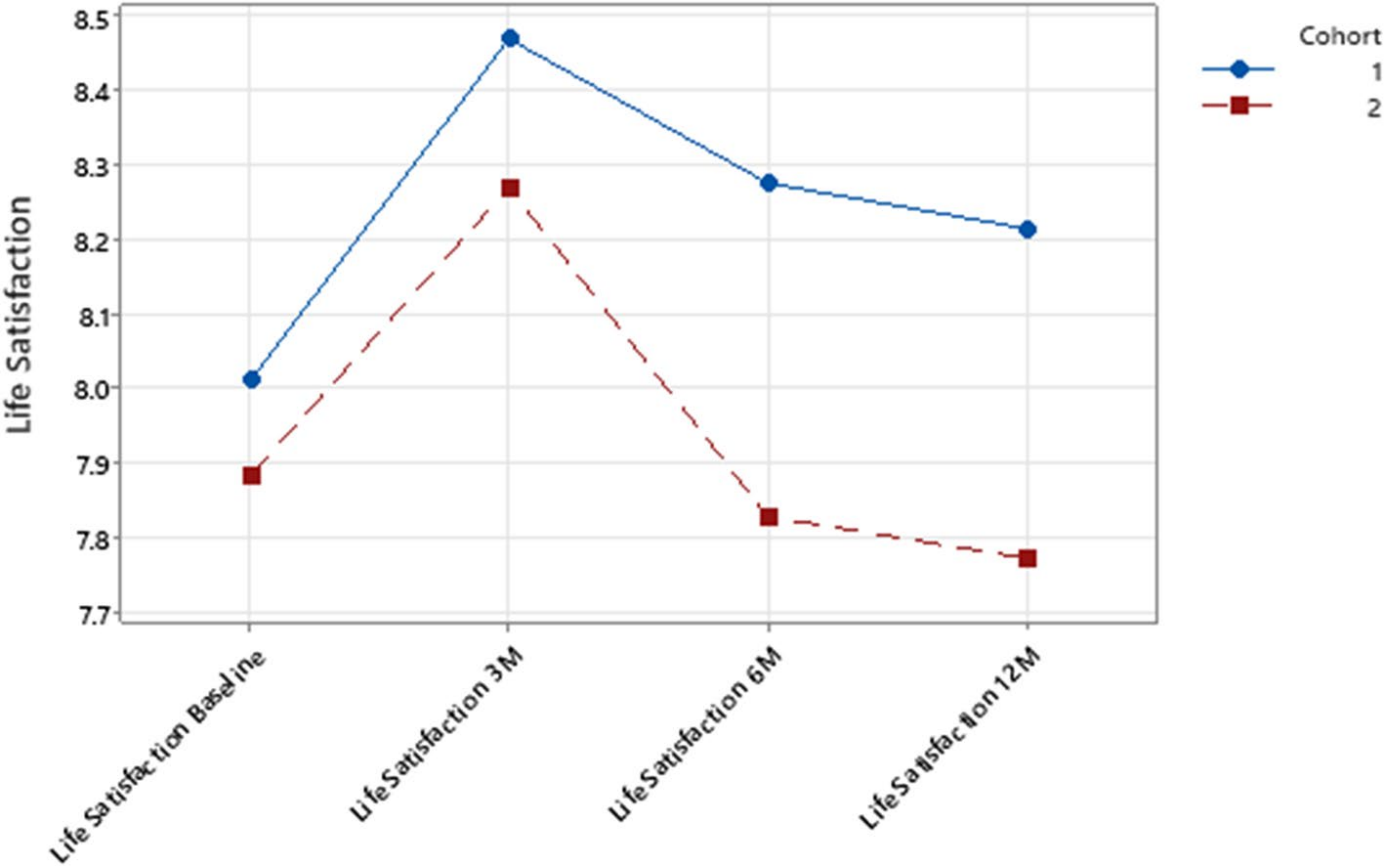
Life satisfaction across time period by cohort. IG = Intervention group C1 = Cohort 1 (pre-COVID cohort) C2 = Cohort 2 (COVID cohort)

Two variables (life satisfaction and SWEMWBS) have notable trends in these analyses.. Firstly, trends for both cohorts for life satisfaction from T1 to T4 are shown in Fig. 1 which highlights that the increase in life satisfaction remained above baseline levels for C1 through to T4 (and the difference between C1 and C2 was significant at T4), suggesting that COVID-19 restrictions and subsequent Shed closures at these time points may have had a negative impact on life satisfaction for C2 (see also Table 2).

**Fig. 2:**
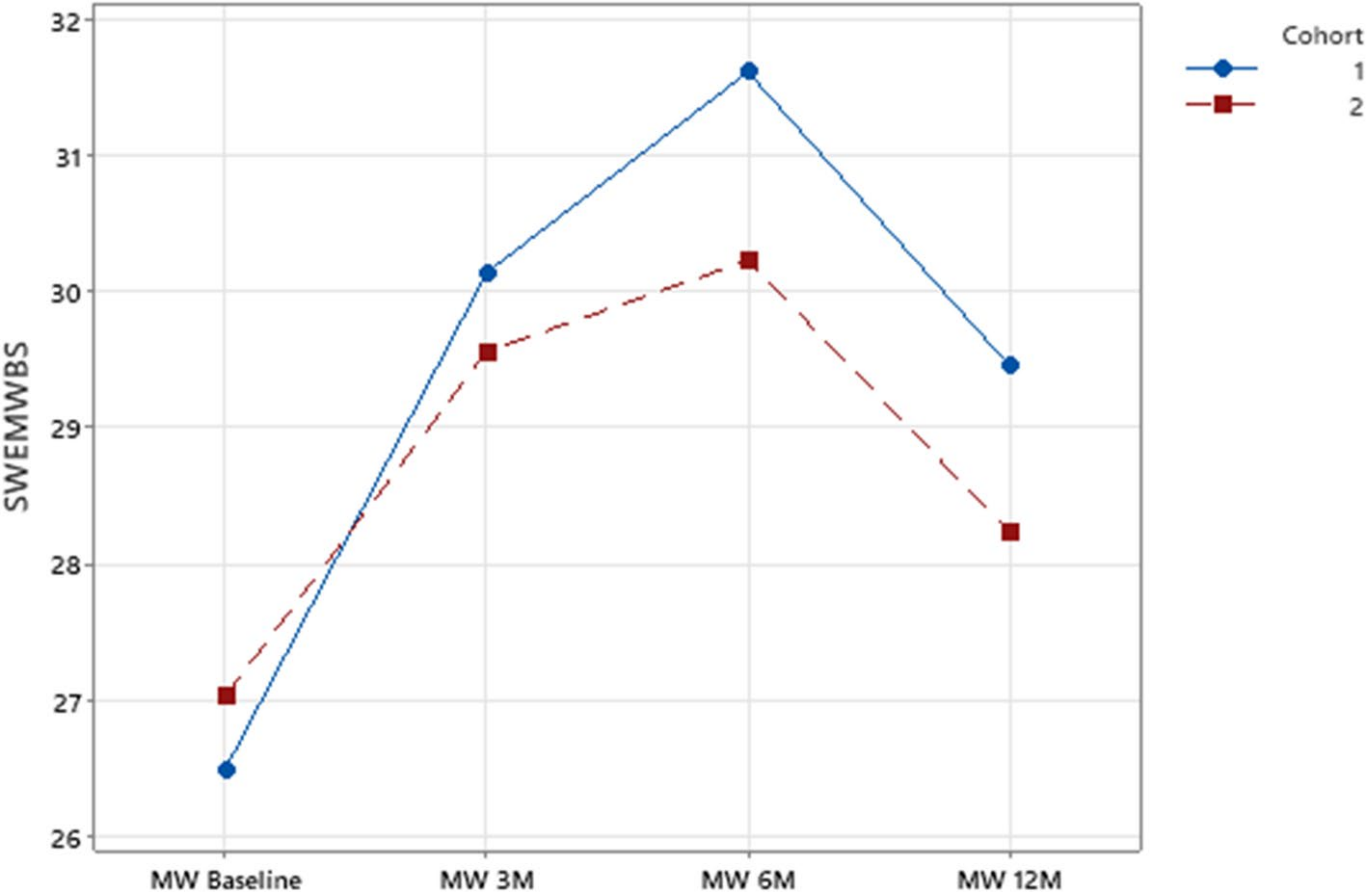
SWEMWBS across time period by cohort. IG = Intervention group C1 = Cohort 1 (pre-COVID cohort) C2 = Cohort 2 (COVID cohort)

Mental wellbeing (SWEMWBS) remained significantly higher for the IG up to 12 months (T4) as shown in Fig. 2. This shows that both C1 and C2 experienced sustained improvements in their mental wellbeing scores; however a significant difference between the two cohorts emerges at T3 and T4 with lower scores in C2, suggesting that COVID-19 may have impacted on overall mental wellbeing.

Depression prevalence remained significantly lower from T1 to T3 and T4 for the IG. This reduction was also observed in both cohorts. There was however a significant difference between depression prevalence scores at T4, highlighting again that C2 may have begun to experience a slight decline in mental wellbeing under COVID-19 restrictions. Significant improvements in certainty about managing mental health between T1 and T2 were maintained up to T4. Despite a slight decline from T1 to T2, shedders levels of comfort in having a conversation about their mental wellbeing remained significantly above T1 values for both C1 and C2.

Significant increases in life worth between T1 and T2 for the IG were sustained up to T4. There were no significant differences between C1 and C2 for life worth. For SRH, Shedders health ratings remained significantly improved at T3 and T4 for the IG. This improvement did not appear to be sustained in C2 with a significant difference between the two cohorts at T3, suggesting COVID-19 may have contributed to this decline.

While SFL did not appear to significantly impact loneliness scores, there was a clear divergence between the two cohorts at T3 and T4. The non-COVID-19 cohort (C1) largely maintained lower loneliness scores while the COVID-19 cohort (C2), experienced a significant increase in loneliness during COVID-19 restrictions and Shed closures, with a significant difference between the two cohorts. In terms of social capital, trust ratings for the IG remained significantly higher than baseline up to T3. While both groups experienced a reduction, trust ratings remained significantly higher for C1 at T4, whereas C2 may have experienced an accelerated decline. Shedders’ sense of belonging remained significantly higher post SFL at T3 and T4. While sense of belonging was largely maintained for both groups, the increase did not remain significantly different for C2 at T4. Similar trends were observed in relation to Shedders’ sense of close support. There was a significant and sustained improvement in close support up to T4 for the IG but the difference did not remain significant for C2 up to 12 months. Mean utility scores continued to increase from T2 to T3 and while there was a slight decline at T4, scores remained significantly higher than baseline for both cohorts up to T4.

### Physical activity and lifestyle

Days physically active remained significantly higher than baseline for the IG and, while there was no significant difference between C1 and C2, this was not sustained for C2. While mean days physically active remained higher at T4 compared to T1 for C1, this cohort did experience a decline. However, C2 experienced a significant increase in days physically active at T4, having previously experienced a reduction. This trend can also be seen in days spent walking where levels remained significantly higher than baseline up to T4, however C2 surpassed C1 for in terms of days walking at T4, suggesting that COVID-19 encouraged an increase in physical activity for C2 during Shed closures.

In relation to diet and cooking habits, fruit and vegetable consumption remained significantly higher up to T4 compared to baseline for the IG in C1 and C2. Positive changes in cooking habits were also sustained post SFL up to T4 for the IG in both cohorts. In terms of cooking frequency, it appears at T3 that the positive increase from T2 was not sustained. However, in C1 this trend had continued in a positive direction with a significant difference being recorded between T1 and T2. The opposite was observed for C2 where this cohort had begun to revert towards baseline ratings. At T4 however, the improvement was again significant for both cohorts. Total cooking confidence scores remained significantly higher for the IG in both cohorts up to T4. There was a significant difference in scores between C1 and C2 at T3 where C2 had declined and scores for C1 continued to increase. Scores became similar at T4 where both groups appeared to maintain confidence levels experienced post SF. Similarly to T2, there was no significant variations in days consuming alcohol. Alcohol units per session remained significantly lower than T1 for the IG in both cohorts however there was a gradual increase from T3 onwards.

### Binary logistic regression

The output from the regression models is shown in Table 3.

**Table 3:** Binary logistic regression analysis for changes in dependent variables (baseline to 3 months)

Dependent Variable	Positive Change in Life Satisfaction		Positive Change in Mental Wellbeing Above Threshold of 2		Positive change in Physical activity 1+ day		Positive change in food preparation & cooking confidence		Positive change in trust	
*Independent Variables*	*Odds Ratio*	95% CI	*Odds Ratio*	95% CI	*Odds Ratio*	95%CI	*Odds Ratio*	95% CI	*Odds Ratio*	95% CI
Age	0.99	0.95–1.01	1.03	0.99–1.07	0.99^a^	0.95–1.02	0.99	0.95–1.02	1.01	0.97–1.04
Education	1.09	0.78–1.54	1.38	0.93–2.06	1.48	0.99–2.19	0.95	0.65–1.37	0.84	0.59–1.18
Living Situation	1.16	0.60–2.25	0.46^a^	0.19–1.06	0.87	0.40–1.89	0.48^a^	0.22–1.05	0.84	0.45–1.63
Days PA at Baseline	1.00	0.90–1.12	0.90	0.79–1.02	0.58^b^	0.50–0.67	0.91	0.81–1.03	0.98	0.88–1.09
Loneliness at Baseline	0.92	0.68–1.25	0.64^a^	0.46–0.92	1.30	0.86–1.99	1.48	097–2.27	0.98	0.73–1.32
Mental Wellbeing at Baseline	0.91^b^	0.86–0.97	0.80^b^	0.76–0.87	1.03	0.96–1.09	1.00	0.94–1.07	0.93^a^	0.88–0.98
Group	5.42^b^	2.65–11.07	17.82^b^	9.76–28.92	5.96^b^	2.93–11.82	9.38^b^	4.72–18.63	3.55^b^	1.82–6.94
N	285		277		280		273		276	

^a^Significant at 5% level

^b^Significant at 1% level Education: 1 = primary education 4 = postgraduate education Living situation 1 = living alone 2 = living with others Group: 0 = Intervention 1 = Control

Using a change in life satisfaction as the dependent variable, a significant relationship with baseline levels of mental wellbeing is found i.e. those with lower baseline levels of mental wellbeing have almost 9% (1–0.911 = 0.089) higher odds of experiencing an improvement in life satisfaction from T1 to T2. An odds ratio of 5.419 also suggests that the IG is over 400% more likely to experience this change compared to the CG. Using a change in SWEMWBS above a threshold of 2 as the dependent variable, Shedders who live alone have higher odds of experiencing a clinically meaningful change in SWEMWBS compared to Shedders who live with others. Shedders with lower loneliness scores however have higher odds of experiencing positive change in SWEMWBS compared to Shedders with higher loneliness scores. Shedders with lower mental wellbeing at baseline have also higher odds of experiencing a meaningful change in SWEBMWS. Shedders in the IG are almost 18 times more likely to experience a positive change in SWEMWBS compared to Shedders in the CG. Using positive change in PA as the dependent variable, younger Shedders have higher odds of experiencing a positive change in their weekly PA. Shedders who were more inactive at baseline are also more likely to increase their weekly PA by one day or more compared to active Shedders. Shedders in the IG are also have over five times more likelihood of increasing their PA compared to the CG. In relation to positive change in food preparation and cooking scores, Shedders who live alone are over twice as likely to experience a positive improvement. The IG are over eight times more likely to experience positive improvements in food preparation and cooking confidence compared to the CG. In terms of positive change in trust, Shedders with lower SWEMWBS have higher odds of experiencing an improvement in trust and the IG was 255% more likely to experience an improvement in trust compared to the CG.

## Discussion

This study describes the impact of a 10-week health promotion programme (SFL) on the health and wellbeing outcomes of Shedders and is the first evaluation of a structured health and wellbeing initiative co-designed and delivered in Men’s Sheds. Results suggest that the gender-specific approach of SFL is effective in engaging cohorts of HTR (older, lower educated, retired, inactive, obese, hypertensive) men within Sheds [34]. Moreover, whilst asking Shedders to opt into SFL might be seen as a potential limitation in terms of how representative the sample was of this cohort, this was offset by a reach rate of almost 75%, indicating that the majority of Shedders opted into SFL. The diverse backgrounds of Shedders may have been conducive towards enriching the learning and engagement of participants, particularly for men who may have been more reticent about participating in SFL [38]. Notably, the majority of participants reported themselves as ‘White Irish’. Whilst this is reflective of the current profile of older men in Ireland [72], due consideration should be paid to encouraging more diversity within Sheds in Ireland and with engagement with SFL [59].

Research indicates that men tend to report lower life satisfaction scores compared to women, but that life satisfaction increases for men in later years [64]. Life satisfaction scores for this older cohort of Shedders were indeed high at baseline (7.98). These were comparable to ratings for men over 50 years in Ireland (7.56) [73] and positively correlated with age at this time point [34]. Younger Shedders were more likely to experience a positive change in life satisfaction at T2 however. The positive increase in life satisfaction in the IG suggests that SFL had a positive impact on life satisfaction. While life satisfaction remained higher than baseline, the trajectory of these scores in C1 suggests that scores began to naturally decline a year later. This decline can also be seen in C2 but results suggests that the impact of COVID-19 restrictions accelerated this decline in C2. Shedders sense of life worth also positively increased following SFL and while it remained higher than baseline 12 months later, a similar trend can be observed where scores begin to level off, yet this did not appear to be impacted by COVID-19. The use of the single-item self-rated health measure is recognised as a reliable way of measuring health despite potential discrepancies in one’s internal view of one’s health misaligning with medical diagnoses [74]. Indeed, while Shedders reported their SRH in positive terms at baseline, this did not align with objective measures of health and suggests that Shedders may prioritise other aspects of wellbeing when evaluating their health [34]. This is an important finding and highlights the importance of the co-design process in SFL and men’s health promotion more broadly, where understanding or priorities for ‘health’ among service providers and Shedders may not always align. Self-rated health did significantly improve following SFL, a change that was sustained a year later for C1 but not C2, suggesting that SFL is capable of having a sustained improvement on SRH outside of COVID-19.

The significant and sustained improvement in those wanting to seek information about their health is a positive indication that the gender-specific approaches which underpinned SFL such as; fostering the non-clinical, safe environment and utilising a strengths-based approach was conducive towards encouraging positive attitudes towards health engagement. Male patients are also more likely to default on appointments than female patients [75]. Considering almost 80% of Shedders were referred to their GP following their health check in the Shed highlights the importance of this intervention to pick up on risk factors that may otherwise go undetected. Of the Shedders referred to their GP, a considerable proportion (41.7%) reported actually following up with their GP at T3. While one would hope to see a majority follow through, considering the cohort of Shedders, this should be considered a positive response.

Similarly to previous research that has focused on engaging hard-to-reach men at community level, particularly ‘Men on the Move’ and ‘Football Fans in Training’ [25, 76], SFL achieved a positive mental wellbeing effect with significant increases in SWEMEBS scores that are considered clinically meaningful in the IG [55]. Those with lower SWEMWBS scores at baseline also experienced the most improvement in mental wellbeing (OR 0.804) life satisfaction (OR 0.911) and trust (0.928), suggesting that SFL had a positive impact in those with poorer mental health. In addition, while there was a marked difference between C1 and C2 in SWEEMWBS at 12 months, results suggest that, despite COVID-19, Shedders retained an improvement in mental wellbeing as well as a sustained reduction in depression prevalence. It is widely accepted that men experience barriers with engaging in conversations about mental health, often exacerbated by social constructs of what it means to be ‘masculine’ [77–79]. This narrative has been challenged by research that highlights that when men are familiar with problem-solving strategies to maintain their mental wellbeing, they are open to using them [80–82]. Indeed SFL mirrors these findings where significant and sustained improvement in Shedders own self-efficacy in relation to managing and talking about mental health, demonstrates the efficacy of the SFL initiative in creating an environment where Shedders can openly discuss and feel supported with their mental wellbeing. It is important to note that while scores for these constructs remained significantly enhanced, scores began to revert at 6 and 12 months which highlights the importance of identifying strategies for Shedders to maintain the benefits gained from the initial 10-weeks of SFL.

The Shed environment is recognised as a setting which promotes social support and protects against isolation and loneliness [34, 83, 84]. This inherent Shed benefit was reflected in Shedders’ lower loneliness scores at baseline and the steep rise in loneliness in C2 at 6 and 12 months following Shed closures during COVID-19 [61]. This suggests that SFL did not impact Shedders’ loneliness significantly as Shedders reported minimal loneliness at baseline possibly due an organic Shed effect. While it appears Sheds may indeed have a protective effect against social isolation, constructs of social capital (trust, belonging and feelings of having close support) also positively improved following SFL. This suggests that SFL further enhanced the sense of social capital in Sheds, which may have been a result of the enhance sense of social cohesion during SFL, where research suggests that Sheds with a primary aim on social participation offer meaningful opportunities for inclusivity of a diverse range of Shedders [35, 37, 85]. Older men who are more vulnerable, such as those who live alone, are at risk of depressive symptoms due to lower levels of sense of belonging [65]. Previous research on Sheds highlighted the importance of the Shed space for older men in reducing isolation aided by access to programmes [30] and the significant increase in those who felt like they belonged to their Shed highlights the potential of SFL to build upon, and further enhance the social support inherent in Sheds. Research also highlights the relationship between social capital and wellbeing in particular its influence on physical activity and health engagement [66, 67]. Alongside the significant improvement in belongingness, SFL participants also experienced a significant enhancement in feelings of close support and general trust, suggesting that SFL had a positive impact on social capital which may have also encouraged engagement with other positive health behaviours and practices within SFL. While improvements in social capital constructs were sustained in large part for C1, they did begin to revert with an observed accelerated decline in C2, again suggesting the need for SFL to devise strategies to maintain positive benefits beyond the 10 week intervention.

The number of Shedders (68.2%) not meeting the recommended PA levels of 30 minutes or more for 5 days per week at baseline was higher than reported in a comparable study ‘the Irish Longitudinal Study on Ageing’ (TILDA) which found that 58% of men over 50 years did not reach the recommended PA guidelines [68]. Older Shedders more likely to meet the PA guidelines than younger Shedders [34]. While again there was some reversion in PA levels, days physically active remained higher than baseline one year later for the IG as did days spent walking. Overall, it appeared that there was a natural reversion in C1 highlighting the need for a maintenance phase in Sheds to encourage sustainment and further improvement of PA levels. In C2 days physically active, and days spent walking in particular, saw an increase at 12 months which may have been in part due to COVID-19 and the limitation of other recreational activities for older citizens beyond outdoor PA [61]. Regression work suggests that those who were less active at baseline were more likely to increase their PA levels (OR 0.582), also highlighting that SFL may have been effective in mobilising more inactive Shedders. Moreover younger Shedders experienced greater improvements in their PA levels. This is a positive finding considering that older Shedders were significantly more physically active at baseline, with research suggesting that younger Shedders may attend the Shed due to poorer health [34]. The significant and sustained improvement in PA self-efficacy is also a positive finding that suggests SFL was effective in enhancing self-efficacy which may be a stronger predictor of sustained engagement with PA compared to self-rated PA, as well as being strongly and independently associated with cardiovascular events in men [86].

Alongside active living, healthy eating is a key priority of the Healthy Ireland Men’s Action Plan with increased morbidity and mortality rates linked to lifestyle based determinants such as eating behaviours [16]. Men are more vulnerable to poor nutrition due to a variety of social determinants such as food shopping, preparation and cooking traditionally organised by women, with advertising, health literacy and health promotion messages related to healthy eating targeted towards, and subsequently engaging, more women [87, 88]. This is particularly the case for more vulnerable men such as those who are older, live alone, or have lower educational attainment [69, 89]. Similar to the HATRICK approach which uses informal environments and social engagement opportunities to deliver messages around healthy eating, while also appealing to practical elements of cooking for men [26], SFL has demonstrated a positive and sustained change in food preparation and cooking confidence. Alongside this, Shedders who lived alone were more likely to experience positive changes in their cooking confidence and food preparation (*OR* 0.481) suggesting that SFL was effective in enhancing outcomes for more HTR Shedders. Moreover, a significant proportion of Shedders (25.8%) were either separated or divorced at baseline, highlighting the utility of the Sheds to attract HTR cohorts of men. The positive outcomes post SFL in relation to healthy eating and cooking behaviours suggest that the Healthy Food Made Easy programme within SFL has been successful in engaging men with messages around healthy eating behaviours and encouraging positive and lasting changes. Less than 10% of Shedders reported drinking more than the recommended 17 standard drinks per week at baseline [34]. Previous studies which seek to engage men note a similar findings which may be in part due to age profile or self-report bias [28]. While there may be in accuracies in self- reporting of alcohol units consumed versus actual consumption, days spent consuming alcohol per week as well as alcohol units reduced significantly following SFL. This change was not sustained with a significant increase in days spent consuming alcohol at 12 months. While SFL did not have a specific focus on alcohol behaviour, overall alcohol consumption and frequency of binge drinking is higher in men than in women with up to 54% of Irish men classified as heavy episodic drinkers and is therefore an important consideration for SFL going forward [16, 90] .

## Conclusions

This research has demonstrated that SFL is an effective model that engages Shedders with health and wellbeing and encourages positive and sustained change in terms of health and wellbeing outcomes such as mental wellbeing, social capital, diet and cooking confidence, subjective wellbeing and physical activity. It highlights the conducive environment of the Shed as a setting in which to activate gender-specific approaches built upon the organic health promotion qualities of the Shed, that effectively engage men in a safe, familiar and informal way while providing opportunities for structured health and wellbeing initiatives through this inclusive, community-based approach. The findings highlight the potential of SFL to improve the health and wellbeing of all Shedders but in particular it’s potential to encourage more positive gains for Shedders who may have been harder to reach at baseline, highlighted by the increased gains made by men who lived alone and with lower baseline levels of mental wellbeing, subjective wellbeing and physical activity. The successful reach of SFL in targeted Sheds is a testament to its potential for scale-up alongside its sustained effect across implementation environments which highlights the capability of the SFL approach to be transferrable across multiple and variable Shed settings. While COVID-19 had an impact on the trajectory of Shedders wellbeing outcomes over the 12 month follow up period, many outcomes were not impacted at a significant level and importantly, Shedders who experienced COVID-19 maintained improvements in mental health despite a significant increase in loneliness as well as improving their physical activity levels. It is important that SFL remains true to its ethos as it evolves over time to respect the environment of the Sheds and continually respond to needs of Shedders, particularly in the wake of COVID-19. The findings highlight the importance of the co-design approach of SFL and for men’s health promotion more broadly where service providers and practitioners should give due consideration to understanding what Shedders prioritise in terms of their wellbeing. While SFL has demonstrated efficacy in engaging HTR men, it also highlights that lonely Shedders are more at risk of poorer mental wellbeing and efforts at engaging more vulnerable Shedders should be prioritised, particularly in the wake of COVID-19 which has clearly exacerbated loneliness. It is also important to highlight that while health outcomes did improve, there was evidence of reversion a year later and it is recommended that the SFL design adapts to incorporate a maintenance phase in order to sustain the positive improvements Shedders gained within the 10-week intervention. The SFL programme has highlighted the potential that tailored and targeted men’s health interventions can have in terms of addressing gender inequalities in health and can inform health promotion strategies in Sheds as well as other community-based settings that engage men with health.

### Limitations

There are clear limitations in this study which should be noted. Firstly, due to capacity constraints at the time of data collection further compounded by the onset of COVID-19, the control group was small in comparison to the intervention group. However, research has demonstrated that there is value in having a small control with a larger intervention group in community-based programmes where there are often capacity constraints [91]. The recruitment of participants into SFL was a sensitive process facilitated by gender-specific approaches where buy-in and trust building is critical to engagement. Therefore, respecting the autonomy of Shedders to opt in/out of the programme on their terms took precedence over any attempts to generate a larger size control group. The advent of COVID-19 also meant that it was not possible to recruit a further control group and the waist list control group received the SFL intervention with concentration on the intervention group for the remainder of the study due to reduced resources. COVID-19 also became a significant confounder in relation to follow up periods made visible by the impact in the health and wellbeing of Shedders [61]. This also removed the potential for multivariate modelling up to 12 months that could accurately capture SFL impact. However, separate analysis of both cohorts helps to limit this impact. The subjective nature of the data and the inherent bias in the self-report format should also be noted, particularly considering the study design where participants are aware they have received an intervention. While the evidence suggests that the recruitment strategy was effective in engaging the target group of Shedders, this approach may lead to a potential selection bias when applied to HTR groups outside of Sheds. Finally, while comparisons can be made between Shedders and the general population of older males in Ireland, SFL is an initiative tailored to the Sheds setting, and therefore, generalizability is limited to the Shedder population.

## Data Availability

The data sets analysed during the current study are available from the corresponding author on reasonable request. A detailed protocol paper can be found at: https://bmcpublichealth.biomedcentral.com/articles/10.1186/s12889-021-10823-8.

## Abbreviations

C1: Cohort 1
C2: Cohort 2
CG: Control Group
CPR: Cardiopulmonary resuscitation training
HFME: Healthy Food Made Easy
HTR: Hard-to-Reach
IG: Intervention Group
IMSA: Irish Men’s Sheds Association
OR: Odds Ratio
PA: Physical Activity
PA: Physical activity
SF-6D: Short Form 6D
SFL: Sheds for Life
Shedders: Men’s Shed members
Sheds: Men’s Sheds
SPs: Service provider organisations
SPSS: Statistical Packages for the Social Sciences
SRH: Self-rated health
SWEMBWS: Short Warwick-Edinburgh Mental Wellbeing Scale
T1: Baseline
T2: 3 month follow up
T3: 6 month follow up
T4: 12 month follow up
TILDA: The Irish Longitudinal Study on Ageing
